# Chinese herbal preparations for chronic heart failure: Study protocol for an umbrella review of systematic reviews and meta-analyses

**DOI:** 10.1097/MD.0000000000018966

**Published:** 2020-02-14

**Authors:** Yong Li, Xiaohua Zhang, Xiaoxiao Chen, Dezhu Chen, Qian Yu, Shenglan Yang, Mingjian Lang

**Affiliations:** a Department of Cardiology, The Fifth People's Hospital affiliated to Chengdu University of Traditional Chinese Medicine; b Jane Lab, Big Data Research Center, University of Electronic Science and Technology of China, Chengdu, China.

**Keywords:** Chinese herbal preparations, heart failure, network meta-analysis

## Abstract

**Background:**

Chinese herbal preparations (CHPs) have been reported to be effective in the management of chronic heart failure (CHF); they are beneficial in improving cardiac function, reducing hospital stays and readmission. However, the credibility of their effectiveness evidence has not been evaluated. We aim to summarize and evaluate current effectiveness evidence of traditional Chinese medicine in the management of CHF.

**Methods:**

We will search PubMed, Embase, the Cochrane Database of Systemic Review (CDSR), and Web of Science from inception to December 2019 for systematic reviews that assessing the effectiveness of CHPs for CHF. The search will be performed without language restriction. Experimental interventions will include any type of CHPs, and control interventions will include placebo, sham interventions, usual care, or no controls. The primary outcome will be the changes in heart function classification defined by the New York Heart Association. Secondary outcomes include left ventricular ejection fraction, Six Minute Walk Test, other efficacy outcomes, and adverse events. We will use *I*^2^ statistics to assess the between-study heterogeneity in each meta-analysis, Eager test to detect publication bias, and the ratio of observed versus expected number of trials with positive findings. We will summarize the evidence and classify them into convincing, highly suggestive, suggestive, or weak.

**Results:**

The results of this study will be published in a peer-reviewed journal.

**Ethics and dissemination:**

No ethical approval and patient consent are required since this study data is based on published literature. The results of the study will be submitted to a peer-reviewed journal.

**Protocol registration number:**

PROSPERO CRD 42019139649 (https://www.crd.york.ac.uk/PROSPERO/#joinuppage)

## 1 Introduction

Heart failure becomes the most important problem of public health with its characteristics of significant morbidity,^[1,2]^ mortality, and healthcare expenditure.^[3,4]^ One of five patients of chronic heart failure (CHF) were re-hospitalized within 1 year or died globally.^[5]^ Among HF patients in Asian regions, there are at least five distinct patterns comorbidities clustered in,^[6]^ improvement of the management of CHF is warranted.^[7–9]^ Although advancements in diagnosis and treatment induce obvious decline in in-hospital mortality, the long-term outcome, readmission rate, and the quality of life of patients with CHF are still high.^[10]^ Data reports that in the US the rate of readmission in patients with CHF within 1 month was 17.6% for the Patient Navigator Program and 25.6% for the medical center,^[11]^ respectively. Therefore, early diagnosis and in-time treatment are needed for reducing the rate of readmission and mortality.

According to the Heart Failure Management Guideline (jointly updated by ACC, AHA, and HFSA in April 2017)^[12]^ and the ESC Diagnosis and Treatment Guideline of 2016 for heart failure,^[13]^ the pathogenesis of heart failure is further clarified, but the effects of different clinical interventions are not fully evaluated,^[14]^ especially in the field of traditional Chinese medicine.^[15]^ Even though Chinese herbal preparations (CHPs) are widely prescribed as adjunctive therapy for CHF in China, the overall quality of the evidence of these CHPs has not been evaluated.^[16]^ Regarding a large body of evidence for CHPs has been published, we aim to conduct an umbrella systematic review to summarize the general effect sizes of the CHPs, assess the risk of bias (ROB) of the evidence, and provide an evidence map for these CHPs.

## 2 Methods

### 2.1 Study registration

The PROTOCOL scheme matches the PRISMA reporting standards. The study protocol has been registered on PROSPERO (https://www.crd.york.ac.uk/prospero) with a unique ID of CRD42019139649.

### 2.2 Eligibility criteria

#### 2.2.1 Type of study

We will include systematic reviews and meta-analyses that investigate the effect of CHPs for the treatment of CHF.

#### 2.2.2 Participants

We will include patients with CHF, and no limitations will be set on the participant's characteristics.

#### 2.2.3 Interventions and controls

We will include any type of CHPs as experimental intervention, we will include placebo, sham procedures, active control, or no treatments (when CHPs are used as adjunctive therapy to usual care) as controls.

#### 2.2.4 Outcome assessments

The primary outcome will be the changes in heart function classification defined by the New York Heart Association. Secondary outcomes include left ventricular ejection fraction, response rate, ADRs/ADEs, LVEF, BNP, CO, SV, 6MWT, LVEDD & LVESD/LVEDV&LVESV, Mortality/death, PaO_2_ & PaCO_2_, etc.

### 2.3 Data source

We will search PubMed, Embase, the Cochrane Database of Systemic Review (CDSR), and Web of Science from inception to December 2019 for systematic reviews that assessing the effectiveness of CHPs for CHF. We will also search PROSERPO (https://www.crd.york.ac.uk/) for relevant reviews. The search will be performed without language restriction. We will also search the websites of the US Food and Drug Administration (https://www.fda.gov/), the American Heart Association (https://www.heart.org/), the European heart association (http://www.heartassociation.eu/), and the European Society of Cardiology (https://www.escardio.org/) for relevant systematic reviews and meta-analyses.

### 2.4 Study selection

Studies that meet the aforementioned eligibility criteria will be considered for further screening. We will exclude studies with any of the following conditions:

(1) duplicated publications;
(2) data are unavailable or incorrect, or no relevant data for meta-analysis;
(3) meta-analysis of quasi randomized controlled clinical trials (defined as allocation using alternation, the sequence of admission, case record numbers, dates of birth), non-randomized controlled clinical trials, or observational studies;
(4) the included participants are diagnosed as CHF with unclear heart function classification;
(5) combined with any other herbal medicines in the control group.

### 2.5 Data extraction

Data extraction will include characteristics of systematic reviews and meta-analyses (first author, publication year, the number of trials included, the number of participants in each meta-analysis, and methods used for pooled analysis), the interventions they received (name, dose, frequency, and the total duration of treatment), the monitoring for efficacy or adherence, and the measure of outcome (specifically defined as event or measure and time frame for the ascertainment of this outcome). For studies with more than one follow-up period, we will select the longest.

### 2.6 Synthesis of included studies

This protocol will summarize the main findings of the eligible systematic reviews. For systematic reviews with meta-analysis, we will use random-effects model (meta package in R 3.5.0) calculate the summary ES and 95%CI. We will estimate the 95% prediction intervals (PIs) and assess whether they excluded null value. We use *I*^2^ statistics to assess the between-study heterogeneity in each meta-analysis. We classified the heterogeneity as three degrees: small (*I*^2^ < 25%), moderate (25% < *I*^2^ < 50%), and large (*I*^2^ > 50%). And use the Egger test to evaluate publication bias and small-study effect. To evaluate the excessive significant bias, we will run a test to assess whether the observed number of studies (*O*) with significant results (positive studies with *P* < .05) is larger than their expected number (*E*). *E* is calculated by the sum of the actual power of each original study, the true effect size of HP infection will be estimated through the parameters of the original study with the largest sample size in a meta-analysis. We will also calculate the *O*/*E* ratio to evaluate the extent of excess significance bias and assess the statistical significance of the bias through chi-squared test; when a *P* < .05 is reached, we will consider the existence of significant bias.

The evidence of the effectiveness of Chinese herbals in chronic heart failure diseases will be categorized into strongest-validity, highly suggestive, suggestive, or weak evidence according to the criteria 21. The evidence with strong-validity will fulfill:

(1) *P*-value < .05 in fixed-effects model or had *P*-value < .001 in random-effects model;
(2) at least 1000 participants;
(3) low or moderate between-study heterogeneity (*I*^2^ < 50%);
(4) 95%PI that excludes the null value;
(5) no evidence of small-study effects and excess significance bias.

The highly suggestive evidence meets criteria (1) to (4); the suggestive evidence meet (1) and (2); the weak evidence will meet only (1).

### 2.7 Methodological quality evaluation

Two reviewers will independently appraise the methodological quality of the included studies using the Measurement Tool to Assess systematic Reviews second version (AMSTAR2). AMSTAR2 is a revised version of AMSTAR, a popular instrument for critically appraising systematic reviews of randomized controlled trials (RCTs). The AMSTAR2 has a total of 16 items to assess seven critical domains of systematic reviews. The seven critical domains include: protocol registered before commencement of the review (item 2); adequacy of the literature search (item 4); justification for excluding individual studies (item 7); ROB from individual studies being included in the review (item 9); appropriateness of meta-analytical methods (item 11); consideration of ROB when interpreting the results of the review (item 13); assessment of presence and likely impact of publication bias (item 15). We will classify the quality of included systematic reviews into one of the four levels: high, moderate, low, or critically low. High confidence refers to systematic reviews without non-critical weakness and systematic reviews that provide accurate and comprehensive summaries of available studies that address the question of interest. Moderate confidence refers to systematic reviews with more than one non-critical weakness. Low confidence refers to systematic reviews with at least one critical flaw. Critically low confidence refers to systematic reviews with more than one critical flaw.

## 3 Discussion

Many studies report that the treatment of heart failure by traditional Chinese medicine have obvious advantages over the improvements of the symptoms,^[17]^ activity tolerance, heart function, or electrolyte balance and the reductions in the incidence of heart failure or the rate of readmission. However, there are many drawbacks in the design and implementation of some RCTs of TCM-related researches. Any neglect of every process may lead to bias, eventually affecting the effect of the evaluation. Therefore, the trials of TCM-related researches of the treatment of heart failure should be strictly conducted in a more ideal environment.^[18]^ We are naturally concerned about the generation of random distribution sequence, the implementation of blind method, the selection of control measures and the evaluation of quality. It is necessary to conduct multicenter, large-sample, high-quality, RCTs with cardiovascular events as the end points for the purpose of accumulating more precise evidence-based medical evidence, finally clarifying the efficacy and safety of the treatment of heart failure by traditional Chinese medicine.

This protocol is designed in adherence to guideline for umbrella protocols and will be conducted and reported strictly according to the PRISMA extension statement.

## Author contributions

Yong Li and Xiaohua Zhang conceived the idea for this study; Xiaoxiao Chen, Dezhu Chen and Qian Yu designed the meta-analysis; Shenglan Yang and Yong Li provided statistical advice and input; Mingjian Lang drafted the protocol. Mingjian Lang and Yong Li reviewed the protocol and provided critical feedback. All authors approved the article in its final form.
